# Mind the Gap! Translating Cartilage Research for Patients and the Public

**DOI:** 10.1177/19476035261508436

**Published:** 2026-11-12

**Authors:** Milan Mohammad, Jonathan J. Bjerre-Bastos, Asger R. Bihlet, Elsa Silva, Ali Mobasheri, Mats Brittberg, Gino M. M. J. Kerkhoffs, Jari Dahmen

**Affiliations:** 1 Centre for Physical Activity Research, 53146 Copenhagen University Hospital – Rigshospitalet, Copenhagen, Denmark; 2 Department of Cellular and Molecular Medicine, Faculty of Health and Medical Sciences, University of Copenhagen, Copenhagen, Denmark; 3 Department of Orthopedic Surgery, Bispebjerg Hospital, Copenhagen, Denmark; 4 660138 NBCD A/S, Soeborg, Denmark; 5 Liga Portuguesa Contra as Doenças Reumáticas (LPCDR), Porto, Portugal; 6 Research Unit of Health Sciences and Technology, Faculty of Medicine, 6370 University of Oulu, Oulu, Finland; 7 Department of Personalized Medicine, Innovative Medicine Center, Vilnius, Lithuania; 8 Department of Joint Surgery, First Affiliated Hospital of Sun Yat-sen University, Guangzhou, China; 9 Department for Health Sciences, Medicine and Research, Center for Regenerative Medicine, Faculty of Health and Medicine, University for Continuing Education Krems, Krems, Austria; 10 Cartilage Research Unit, Region Halland Orthopaedics, Varberg Hospital, University of Gothenburg, Varberg, Sweden; 11 Department of Orthopedic Surgery and Sports Medicine, Amsterdam Movement Sciences, Amsterdam UMC, Location AMC University of Amsterdam, Amsterdam, the Netherlands; 12 Academic Center for Evidence-based Sports Medicine (ACES) Amsterdam UMC, Amsterdam, the Netherlands; 13 Amsterdam Collaboration for Health & Safety in Sports (ACHSS), International Olympic Committee (IOC) Research Center Amsterdam UMC, Amsterdam, the Netherlands

**Keywords:** Osteoarthritis, Health communication, Social media, Patient participation, Infodemiology

## Introduction

Osteoarticular disorders that affect cartilage structure and function, including traumatic cartilage lesions and osteoarthritis, impose a major burden on both individuals and society.^1,2^ For patients, the consequences are rarely abstract: pain, fatigue, functional limitation and progressive constraints on daily life are common,^3^ affecting their psychological well-being, social life, and ability to work.^4-6^ This context helps explain why patients closely follow new studies and why expectations can escalate when research findings are not communicated sufficiently.

In parallel with this burden, cartilage science and clinical practice are evolving rapidly. Cartilage is a specialized tissue with limited intrinsic repair capacity, whose function depends on the structural integrity and tightly regulated turnover of its extracellular matrix. Accordingly, symptomatic improvement or changes in biochemical biomarkers and imaging parameters should not, in isolation, be interpreted as evidence of functional tissue restoration or true cartilage regeneration. Against this biological background, the field now spans biomarker discovery, increasingly sophisticated imaging, and a growing therapeutic repertoire ranging from rehabilitation and injectable therapies to marrow stimulation, osteochondral procedures, and chondrocyte-based repair strategies.^7,8^ This has gone hand in hand with clinical development, which has led to improved surgical options and crucial pharmacological discoveries.

It is thus unsurprising that patients and their families increasingly ask, often explicitly, what new research findings mean for them.^9^ This is a reasonable question and, in an editorial sense, a necessary one, in order to reduce anxiety and pain.^5^ Decisions in healthcare, policy, education and other fields use data to drive action and change. Communicating research findings from researchers to the public is therefore essential if knowledge acquired is to assist with advancing a field, changing practice and policy, or learning from the challenges and barriers encountered during a study. However, while physicians and researchers know what they know, they are accustomed to communicating mainly in a scientific language and in academic forums, and less often, or not at all, to broader audiences. The risks include that research becomes inaccessible or selectively interpreted, effectively resulting in misinformation.

If cartilage research is indeed evolving and approaching breakthroughs, then the need to mind the gap between researchers and public becomes more urgent. The credibility of research will depend not only on methodological rigour and innovation, but also on the discipline of communicating what is known, what is plausible, what remains uncertain, and what is not yet ready for routine care. Bridging this gap is fundamental to aligning expectations with evidence, sustaining mutual public trust, and ensuring that the knowledge generated by research can responsibly inform patients, practice, and policy.^10^ Accordingly, this editorial focuses on focal chondral or osteochondral lesions and osteoarthritis at different stages, as these clinical scenarios differ substantially in disease biology, treatment goals, and the evidence supporting cartilage repair or regeneration.

## The Communication Gap in the Digital Era

The communication gap in the digital era has created a challenge for researchers, as the rapid proliferation of social media and health misinformation has enabled commercially driven or oversimplified narratives about “regeneration”, including stem cell therapies, PRP (platelet-rich plasma), other orthobiologics, and scaffold-based cartilage repair, to dominate public discourse, often outpacing evidence-based scientific communication. This environment threatens patient safety and the integrity of legitimate research. For example, the direct-to-consumer marketing of unproven stem cell therapies for cartilage and musculoskeletal conditions has reached epidemic proportions. An analysis of 896 practice websites advertising stem cell therapy found that 95.9% contained at least one statement of misinformation, with an average of 4.65 misleading claims per site.^11^ These false claims ranged from errors in basic stem cell biology to outright fabrications about clinical effectiveness, including unfounded promises of cartilage regeneration, inaccurate descriptions of mechanisms of action, and scare tactics designed to pressure patients into treatment.^11^ They present a false alternative truth, constituting a public health problem that must be combated without aggravating the suffering of patients and their families.^6,12^

Regenerative medicine must be led by medical professionals grounded in research, regulation, and accountability - not by market forces. As an example, the question is not whether stem cells hold promise, but who should responsibly guide their translation into patient care. Social media platforms have amplified this problem dramatically, as they offer easier solutions to common issues in cartilage disorders.^13^ Analysis of Instagram and Twitter (now X) posts related to cellular therapy for musculoskeletal conditions revealed that of all posts, 83% originated from businesses or organizations, 94% had an exclusively positive tone and 68% were advertisements.^14^ Notably, approximately half of these advertising posts came from a single business that recruited physicians to market cell-based therapies on social media using multiple hashtags to enhance visibility.^14^ This creates an echo chamber where misinformation is systematically amplified while balanced, evidence-based information struggles to compete. This is not restricted to Instagram and Twitter (now X), but can be expected on professional platforms, such as LinkedIn, as well. YouTube and other video platforms present similar challenges, with algorithm-driven content promotion allowing low-quality, misleading information about recent advances in cartilage research treatments to outpace evidence-based guidance.^15^ This is particularly problematic in cartilage research, where changes in biochemical markers, imaging parameters, or clinical symptoms may be incorrectly presented as evidence of structural cartilage regeneration.

AI (artificial intelligence), especially LLMs (large language models), is now also increasingly becoming a tool through patients access and interpret cartilage-related information. These tools may improve accessibility by summarising technical evidence and adapting explanations to different levels of health literacy. However, LLM-generated responses may be inaccurate, outdated, biased, or insufficiently referenced, and may reproduce the same overinterpretation of symptomatic, imaging, or biomarker outcomes discussed above. Early evaluations of chatbot responses to hip and knee osteoarthritis questions have demonstrated that recommendations are not yet consistently aligned with clinical evidence. Accordingly, AI-generated cartilage information should include transparent sources, clearly communicate uncertainty and treatment limitations, and remain subject to expert oversight.^16-18^

Misinformation does not spread in a vacuum but is shaped by both structural and socioeconomic barriers to care. When evidence-based cartilage treatment is costly, poorly reimbursed, difficult to access, or delayed by limited specialist availability and long waiting times, patients may be more vulnerable to commercially persuasive narratives that offer hope and apparent access. Misinformation is therefore not only a communication problem, but also a manifestation of social inequity, becoming more appealing where evidence-based care is less attainable. The consequences of this misinformation are profound. A survey of 931 patients in the U.S. found that 84.5% believed stem cell therapies have convincing evidence to support their use for orthopaedic conditions, and 65% incorrectly believed these therapies are approved and regulated by the FDA.^19^ Over half of respondents believed stem cells can cure any kind of arthritis, and 63.3% stated they would consider stem cell therapy if their doctor recommended it, regardless of evidence supporting effectiveness.^19^

In contrast to rigorous peer review in scientific publishing, social media platforms have subsequently become a dominant venue for discussing and promoting unproven stem cell treatments. These channels lack systematic evaluation, accountability, and expert oversight, enabling anecdote and marketing to overshadow evidence. True scientific progress depends on transparent, critical appraisal and not the unfiltered echo of social feeds. To counter this misinformation ecosystem, cartilage researchers should communicate proactively using evidence-based strategies. A systematic review of interventions to combat health misinformation identified three key strategy categories^20^: Communication strategies (including myth-busting messages, amplifying factual information, and filling information voids), technology-based strategies (such as credibility badges and algorithmic interventions), and multimedia strategies (including visual abstracts and short videos).^20^ Meta-analytic evidence demonstrates that corrections of health misinformation on social media are effective. Interventions are particularly effective when misinformation is debunked by experts rather than non-experts.^21^ It should be approached with empathy, exploring the emotions underlying belief in information that is not based on evidence or is incorrect, rather than confronting or rejecting it.^10^ Source credibility information and social norms have also been shown to improve truth discernment and reduce engagement with misinformation online.^22^

When scientific papers are presented on social media without context, limitations, or critical appraisal, nuance is often replaced by narrative. Statistical associations become “breakthroughs”. Preliminary findings become “proven treatments”. Caution disappears. Making science accessible must not mean making it unquestionable. A clear explanation for the public should still reflect uncertainty, methodological limits, and the need for replication. Otherwise, we risk transforming careful research into persuasive messaging and turning communication into promotion. In order to ensure robust evidence is heard over hype, cartilage researchers should consider implementing a comprehensive communication strategy for communicating their research findings that includes: (1) Tailoring messages to specific populations using demographic, geographic, and interest-based targeting; (2) including members of target communities in message development to ensure cultural sensitivity and trust-building; (3) directly addressing misinformation through myth-busting messages that highlight false claims and reiterate accurate information; (4) leveraging information sharing by designing shareable content and partnering with trusted influencers; and (5) evaluating impact by measuring real-world outcomes beyond simple engagement metrics.^23^ Podcasts originating from the international osteoarthritis research society are a great example of knowledge dissemination with experts curating credible sources targeting professionals as well as patients.^24^

Overreliance on digital platforms risks widening the communication gap - many elderly patients most affected by osteoarthritis and chronic musculoskeletal disorders are not easily reached through social media or AI-driven content. Equitable cartilage communication must therefore include non-digital, low-barrier channels, especially for older and socioeconomically disadvantaged populations. Importantly, communication success should not be defined by engagement alone. More relevant outcomes in patient care include patient understanding, recognition of uncertainty, decisional confidence, and support for informed treatment choices. Shareable content is only an output, and what maybe matters more is whether patients are better informed and less vulnerable to misleading claims.

## Benefits to Cartilage Researchers and Clinicians

Contrary to the notion that public engagement dilutes academic quality, evidence demonstrates that the engagement in public outreach activities has no significant negative effect on researchers’ performance.^25-27^ Public and patient involvement (PPI) in research can also enhance both the quality and relevance of studies.^28,29^ This is especially pertinent in cartilage research, where treatment choices are highly preference-sensitive and outcomes are judged not only by imaging or structural repair, but also by pain, function, return to activity, treatment burden, and expectations regarding recovery. Clear communication is therefore needed to explain how biochemical markers of extracellular matrix turnover and imaging-based outcomes relate, or may not relate, to clinically meaningful improvements for patients.^8^ Involving patients can therefore help researchers frame questions that better reflect clinically meaningful endpoints and help clinicians communicate why lesion size, symptom pattern, rehabilitation demands, and concomitant pathology matter for treatment selection. Moreover, PPI can significantly accelerate clinical trial recruitment. A systematic review and meta-analysis found that patient and public involvement interventions increased the odds of participant enrolment in clinical trials, with the involvement of people with lived experience of the condition under study being significantly associated with improved enrolment.^30^

Integrating the patient’s voice into scientific research and investigations has evolved in recent years from a simple consultation to a true partnership, as demonstrated by collaborative initiatives such as OMERACT (Outcome Measures in Rheumatology) or PARE (People with Arthritis/Rheumatism in Europe) from EULAR (European Alliance of Associations for Rheumatology), resulting in the development of various co-created interventions and culturally translated and adapted educational resources.^10^ In osteoarthritis, the OMERACT–OARSI (Osteoarthritis Research Society International) core domain set prioritises pain, physical function, quality of life, patient global assessment, and adverse events, while joint structure is included when relevant to the intervention. In cartilage repair, ICRS (International Cartilage Regeneration & Joint Preservation Society) has issued recommendations for PROMs (patient-reported outcome measures) and established a global registry for the longitudinal collection of clinical and patient-reported outcomes.^31,32^ However, collecting PROMs is not equivalent to patient partnership. Patient partners could contribute to setting research priorities, selecting outcome domains and follow-up time points, defining meaningful improvement and acceptable treatment and rehabilitation burden, and interpreting discrepancies between structural or biochemical findings and patient-experienced benefit.

Translating complex concepts for lay audiences forces researchers and clinicians to clarify mechanisms, assumptions, and limitations, a phenomenon related to the “protégé effect” in educational psychology.^33^ When experts explain concepts to novices, they must organize their knowledge in more cohesive and accessible ways, which can enhance their own understanding and identify inconsistencies in their reasoning.^34^ Research demonstrates that the process of preparing to teach or explain material to others engages retrieval practice and elaborative processing, which strengthens the explainer’s own knowledge and reveals areas of uncertainty.^35^ The discipline of science communication may thus serve as a valuable form of intellectual quality control for researchers.

Grant writing and public engagement activities are interconnected forms of science communication that are essential for research to function and benefit society.^36^ Scientists rely on competitive grant funding to finance their research, and this represents an opportunity to communicate their scientific vision and engage funders who are major players in defining how public funds and government policies are prioritized to drive research and innovation.^36^ Researchers consistently regard public engagement as an important and rewarding aspect of research, teaching, and institutional responsibilities, with the potential to enhance public understanding, acceptance, and societal impact.^37^

## Benefits to Patients and the Public

Research participants consistently value receiving research results and learning how their data contributions are enabling health discoveries. A systematic review found that most participants expected researchers to share results regardless of study outcomes, and many participants viewed receiving results as essential for fostering trust with researchers, feeling valued for their contributions, and fulfilling ethical obligations.^38^ Many researchers saw sharing results as a moral duty, especially when participants had limited access to scientific knowledge. The impact of dissemination on research findings included improving health literacy, increased understanding of research, and trust in research.^38^ An American survey found that receiving research results would be valuable to most participants (78.5%) and would make them more likely to trust researchers (70.3%).^39^ Respondents highly valued results revealing information about nearby clinical trials and updates on how their data were used.^39^ Another study found that 60.2% reported that receiving study results increased their willingness to participate in future studies, and 97% agreed that it is very important for researchers to share study results with participants.^40^

A scoping review reported that participants evaluated receipt of trial results positively, with reported benefits including improved communication, demonstration of appreciation, improved retention, and engagement in future research.^41^ However, less than a third of trials currently share results with participants, despite the desire of participants to receive them.^41^

This underscores the importance of proactively communicating research results to participants as a means of building trust, rebuilding psychological safety, enhancing research literacy, and fostering ongoing engagement with the research enterprise.^10^ This is particularly important in a field where patients may be vulnerable to exploitation by unregulated clinics and where trust in legitimate research is essential for advancing evidence-based therapies.

## Challenges

Cartilage biology, imaging, repair techniques, and surrogate endpoints are technically dense, and there is a real risk of creating “false hope” if narrative flow and oversimplified interpretations are prioritised over scientific rigour. Managing public expectations and promoting public understanding about the promise and reality of medical research is an age-old challenge, but the complexity of modern science and the seeming proximity of powerful new approaches to improving human health have heightened the need for the academic community to address this challenge effectively.

Misconceptions in new research findings in cartilage treatment deserve particular attention. For example, favorable extracellular matrix deposition or histological repair in experimentally induced animal models should not be equated with cartilage regeneration in human osteoarthritis. Even when structural effects are observed in clinical studies, these may not translate into symptomatic improvement or demonstrate durable restoration of the architecture and mechanical function of native articular cartilage.^42,43^ Likewise, changes in cartilage-related biomarkers such as CTX-II, COMP, or aggrecan fragments may indicate altered extracellular matrix turnover rather than new cartilage formation. Such changes should therefore not, in isolation, be interpreted as evidence of cartilage regeneration.^8,44^ Public narratives often blur the distinction between symptom relief and structural repair and imply that regenerative interventions have comparable effects in focal cartilage lesions and in early or advanced osteoarthritis, despite important differences in disease biology, treatment goals, and supporting evidence. In reality, contemporary cartilage repair is indication-sensitive, and outcomes depend on lesion characteristics, the joint environment, concomitant pathology, rehabilitation, and the biological quality of the repair tissue. Changes in symptoms, biochemical markers of matrix turnover, or imaging findings should therefore not automatically be interpreted as evidence of functional cartilage restoration or true regeneration.^8,45^

Similar risks of overinterpretation extend beyond stem cell therapies. Symptomatic improvement following PRP or other orthobiologic injections should not be presented as evidence of cartilage regrowth, while outcomes of scaffolds and ACI (autologous chondrocyte implantation) or MACI (matrix-induced autologous chondrocyte implantation) in selected focal cartilage defects should not be extrapolated to diffuse human osteoarthritis.^45-47^ Likewise, although AI-based tools may assist in detecting or grading cartilage abnormalities on imaging, algorithmic performance does not by itself establish clinical validity, generalisability, or an autonomous diagnosis.^48^

Researchers are trained to be sceptical and not overstate existing evidence. Therefore, to be effective in public communication, one must adjust one's mindset and embrace brevity, clarity, and other principles of effective communication, while focusing messages on what is known rather than what is still unknown. However, the implementation of principles of effective communication does not inherently conflict with maintaining scientific accuracy and acknowledging uncertainty, but it requires additional care, effort, and training.^49^

The spread of misinformation in the health field has been associated with higher levels of anxiety, depression, and emotional exhaustion among social media users. Additionally, the use of emotional appeals in this type of content, as well as the emotional state of individuals at the time of exposure, influences the degree of credibility attributed to the misinformation. These appeals tend to divert attention from a more rational evaluation of the information presented. Both positive emotions (such as joy) and negative emotions (such as anger), as well as feelings of social exclusion, can increase susceptibility to accepting misinformation.^10,13,50^

Social media and search algorithms repeatedly expose patients to confident but weakly evidenced claims such as “stem cells rebuild cartilage”. This repetition is particularly dangerous because of the illusory truth effect; repetition alone can make statements feel true, regardless of their actual veracity.^51^ Critically, repetition even increases belief in claims that are implausible or that contradict prior knowledge. To counter the illusory truth effect, cartilage researchers should both “debunk” (reactive) and “prebunk” (proactive) common myths with accurate messages.^52^

Clinicians and researchers who challenge popular but unproven cartilage interventions may face online criticism, trolling, or harassment, with significant effects on wellbeing and public trust in science. A survey of physicians and biomedical scientists found that 66% reported harassment on social media, with 88% of those reporting harassment due to advocacy activities.^52^ Those using social media to post public health messages were significantly more likely to report online harassment. At the same time, most cartilage researchers and clinicians have little formal training in media or digital communication. While medical education focuses on doctor-patient communication at the individual level, the need to train physicians to communicate science effectively to the public is largely ignored.^53^ Scientists are trained to communicate research in technical terms but often lack the skills required to translate findings for broader audiences; yet communication and research translation are increasingly important competencies with wider benefits to science and society.^54^

Ironically, attempting to directly debunk misinformation on social media by making comments on false or misleading claims, is likely to inadvertently increase the reach of the misleading post. This is a result of the current social media algorithms, which favour posts with more interaction and debate, and as a result, the more controversy caused by a post and/or comment(s) made on a post, the more likely it is that the post will be pushed and exposed to a wider audience. Practically, this may be mitigated by researchers or clinical experts not interacting with the misleading post itself but instead make a solid debunking post with no direct hyperlinks to specific misleading posts but rather present a separate critical comment with verbal references to the problematic content, with a format similar to a classical scientific debate, while keeping the receiving audience in mind.

Academic and clinical reward structures rarely recognize public-facing work, which discourages especially junior colleagues from investing time in science communication. A UK-based survey of academic scientists found that while respondents consistently regarded public engagement as an important and rewarding aspect of research, teaching, and institutional responsibilities, enthusiasm was tempered by persistent barriers, including academic workloads, inadequate resources and support, and a lack of formal recognition within career progression.^37^ Respondents emphasized the need for systemic reforms to enable greater participation, including tailored training, sustained funding, and institutional frameworks that acknowledge and reward engagement.^37^ Time constraints, lack of opportunity, and lack of funding are the main barriers preventing researcher participation in public engagement, and joining an existing public engagement program can be a good way for scientists to overcome these barriers.^55^ However, responsibility for translating cartilage research should not rest solely with researchers and surgeons. Pharmacists, physiotherapists, primary care physicians, nurses, and other allied health professionals are often the first or most consistent point of contact for patients with joint and degenerative musculoskeletal disorders. They play a key role in correcting misconceptions, contextualising emerging evidence, and reinforcing realistic expectations regarding rehabilitation, symptom management, and referral pathways. Accordingly, communication training and evidence translation should be embedded across the multidisciplinary workforce.

## Conclusion

Public communication about cartilage research must begin with a distinction that is often lost: treating a focal cartilage defect is not the same as treating osteoarthritis. A traumatic defect may be suitable for cartilage repair in a selected patient. Osteoarthritis, however, affects the joint as an organ; addressing the cartilage surface alone does not address the whole disease. We can repair cartilage defects, but we should be careful with the word regeneration. Recreating normal, durable articular cartilage remains a dream goal, not a routine clinical achievement.

Subsequently, to help robust evidence be heard over hype, the cartilage community should support communication training, recognise public engagement, assist colleagues facing harassment, and respond clearly to misinformation. Every public-facing message should say which condition and patient group a finding concerns, whether a treatment is established or investigational, and what it actually entails, including other procedures, rehabilitation and uncertainty. Above all, results from focal cartilage repair should not be presented as evidence of a cure for osteoarthritis. Successful translation into clinical practice therefore requires evidence that is specific to the cartilage disorder and patient population and that links structural or biochemical effects to durable improvements in symptoms and function. Scientific credibility is not crowdsourced. Rather, it is tested, challenged, and sometimes dismantled through rigorous peer review. If influence replaces scrutiny, we risk allowing algorithms and not evidence to shape clinical practice.
